# Relationship between Changes in Serum Sodium Level and Seizures Occurrence in Children with Hypernatremic Dehydration

**Published:** 2013

**Authors:** Farhad HEYDARIAN, Arezou REZAEIAN

**Affiliations:** 1 Research Center for Patient Safety, Faculty of Medicine, Mashhad University of Medical Sciences, Mashhad, Iran; 2 Pediatric Ward, Ghaem Hospital, Mashhad University of Medical Sciences, Mashhad, Iran

**Keywords:** Hypernatremia, Seizure, Dehydration, Children

## Abstract

**Objective:**

To assess any relationship between serum sodium changes and seizure occurrence in children aged 2 months to 5 years with hypernatremic dehydration.

**Materials & Methods:**

This cross-sectional study was performed on 63 patients aged 2 months to 5 years from 20 March 2006 to 15 March 2012 at Ghaem Hospital and Dr. Sheikh Hospital in Mashhad, Iran. Patients were divided into 2 groups: case group with hypernatremic dehydration and seizure occurrence, and control group with hypernatremic dehydration and no seizures.

**Results:**

The mean age of patients was 10.38 (2-48) months. Thirteen patients had seizures, 11 out of them, before admission and 2 during hospital staying. Serum sodium level at admission in those 2 patients with seizure occurrence after hospitalization was 169 (158-180) mmol/L, and in 50 patients without seizure was 162.8 (148-207) mmol/l. Also, the rate of decrease of serum sodium levels in these 2 cases within the first 12 hours after admission was 1.12, and in those without seizure was 0.54 (mmol/L/hour), and it was 0.47 and 0.53 (mmol/l/ hour) after 24 hours of admission, respectively. Severe dehydration was seen in 38.5% of cases and 14% of controls.

**Conclusion:**

There was not any relationship between changes in serum sodium level and seizure occurrence in children with hypernatremic dehydration.

## Introduction

Seizures occur in children commonly and some studies are performed concerning their different contributing factors (1-5).

Hypernatremic dehydration can be seen with gastroenteritis. Seizures may occur as a complication of the treatment of hypernatremic dehydration. It has been suggested that the rate of decreasing of serum sodium levels should be maintain between 10-15 mmol/L/24hr (6-8). Cerebral edema and seizure can be consequences of rapid correction of serum sodium level in these patients in whom the rate of fluid and sodium administration are inappropriate (9-11).

In one study (12), the relationship between cerebral edema occurrence and rapid rate of fluid administration were detected. 

Another study (13) demonstrated that seizures occurrence is associated with rapid decrease in serum sodium levels at 24 hours after admission. 

One other study (6) showed that there was no relationship between seizures occurrence during hospital staying and the rate of decreasing of sodium during treatment of patients.

Also, in another study (14), which was performed on adult patients, it was concluded that there was not any relationship between dysnatremia complications consisted of neurologic disturbances and rate of changes in serum sodium Considering the importance of seizure occurrence during hypernatremic dehydration in children and also due to some contradictory results of previous studies, we conducted this study to evaluate the relationship between seizure occurrence and serum sodium level changes in children with hypernatremic dehydration.

## Materials & Methods

The present study was conducted as a cross-sectional study on 63 patients aged 2 months to 5 years from March 2006 to March 2012 at Ghaem Hospital and Dr. Sheikh Hospital, Mashhad, Iran. Patients divided into 2 groups: cases with hypernatremic dehydration and seizure occurrence before admission or after hospitalization and controls with hypernatremic dehydration without seizure. Data consisting of age, gender, severity of dehydration, serum sodium levels at admission, 12 and 24 hours after hospitalization, seizure occurrence, andthe rate of sodium and fluid administration were recorded. The amount of administered sodium included those patients who took ≤60 mEq/L or those who received>60 mEq/L in 24 hours. The administered fluid was consisted of those patients who took ≤1.5 times maintenance and those patients who received>1.5 times maintenance in 24 hours.

Hypernatremia was considered when serum sodium level was above 145 mmol/L. The inclusion criteria were patients aged 2 months to 5 years old with gastroenteritis related hypernatremic dehydration. 

Exclusion criteria were severe malnutrition, meningitis, hypoglycemia, hypocalcemia, hypomagnesaemia, neurodevelopment delay, congenital heart disease, any history of epilepsy, and incomplete data in records. 

This study was approved by the Ethics Committee of Mashhad University of Medical Sciences, and was performed according to the principles of the Helsinki Declaration.

Data were analyzed through SPSS software version 16. Quantitative and qualitative data were analyzed by Mann-Whitney and Chi-square tests, respectively. A value of p<0.05 was considered statistically significant for all tests.

## Results

Thirty-one (49.2%) patients were male and 32 (50.8%) were female. The mean age of patients was 10.38 months (2-48). Thirty-nine (62%) patients had moderate dehydration, 12 (19 %) had mild dehydration, and 12 (19%) had severe dehydration. The mean sodium level at admission was 163.1 (148-207) mmol/L and it was 151.2 (139-177) mmol/L, 24 hours after hospitalization. 

In Table 1, some data of patients with seizures and without seizures are shown. 

In Table 2, some data of patients with dropping of serum sodium levels below or above 0.6 mmol/L/h are shown. Two out of 13 patients with seizure had seizure occurrence after hospitalization. Comparison of serum sodium level changes between these two patients and patients without seizure are shown in Table 3.

**Table1:** Some Data Of Dehydrated Hypernatremic Patients With Seizures and Without Seizure

		**With seizures (13 patients)**	**Without seizures (50 patients)**	**p-value**
Gender	Male female	8 (61.5%) 5 (38.5%)	22 (44%) 28 (56%)	0.32
Age (month)		9.5	9.2	0.72
Weight (gram)	At admission	7323.85	7556.2	0.78
	At discharge	7590	7879.6	0.73
Dehydration Severity	Mild Moderate Severe	0 (0%) 8 (61.5%) 5 (38.5%)	12 (24%) 31 (62%) 7 (14%)	0.01
Serum sodium levels (mmol/L)	At admission 12 hours post admission 24 hours post admission	164.3 (151-182) 156 (148-175) 150.7 (139-168)	162.8 (148-207) 153.1 (134-202 147.7 (139-177)	0.68 0.92 0.81
Rate of sodium drop (mmol/L/hr.)	During first 12 hours During first 24 hours > >0.6	0.69 0.59 5(41.7%)	0.55 0.53 16 (41%)	0.29 0.58 0.87
Rate of sodium administration (mmol/L)	≤60 >60	9 (69.2%) 4 (30.8%)	38 (76%) 12 (24%)	0.63

**Table 2:** Comparison of Rate of Dropping of Serum Sodium Levels Below or Above 0.6 mmol/L/h

		**<0.6 mmol/L/h** **(32 patients)**	**≥0.6 mmol/L/h** **(21 patients)**	**p-value**
Serum sodium level (mmol/L)	At admission	159.6 (148-183)	171.4 (156-207)	0.01
	24 h after admission	150.6 (139-170)	152.2 (139-177)	0.42
Mean serum sodium (mmol/L)	During first 24.h	155.1 (139-183)	168.8 (139-207)	0.03
Rate of fluid administration	≤1.5 times maintenance	18 (59.4%)	10 (47.6%)	0.44
	>1.5 times maintenance	13 (40.6%)	11 (52.4%)	
Rate of sodium administration (mmol/L)	≤60	23 (72%)	14 (66.7%)	0.93
	>60	9 (28%)	7 (33.3%)	
Seizure occurrence	After admission	2 (6.3%)	___	0.97
	Before admission	5 (15.6%)	5 (23.8%)	
	Without seizure	25 (78.1%)	16 (76.2%)	

**Table 3:** Comparison Between Serum Sodium Level Changes in Patients with Seizures After Admission and Patients Without Any Seizure

		**With seizures** ** (2 patients)**	**Without seizure** **(50 patients)**	**p-value**
Serum sodium level (mmol/L)	At admission	169 (158-180)	162.8 (148-207)	0.5
Mean serum sodium level (mmol/L)	In 24 hours of admission	160.6 (147-180)	156.8 (134-207)	0.75
Rate of sodium drop (mmol/l/hr)	In 12 hours of admission	1.12	0.54	0.09
	In 24 hours of admission	0.47	0.53	0.86
Sodium administration (mmol/L)	>60	1 (50%)	12 (24%)	0.80

## Discussion

According to the findings of our study, there was no relationship between serum sodium level changes and seizure occurrence in patients with hypernatrmic dehydration.

In a study (13) performed on 48 patients who had hypernatremic dehydration, it was detected that mean serum sodium level at admission was 163.8 mmol/L and the mean serum sodium fall, 6 and 24 hours after admission was 0.54 and 0.52 mmol/L/h, respectively. 

Also, it was revealed that 3 (6.3%) patients had seizures at hospital with dropping of serum sodium levels to 0.51 in group without seizure and to 0.63 in group with seizures (p=0.037). These 3 patients with seizures occurrence had taken more fluid as initial therapy than those patients without seizures (40 cc/kg normal saline vs. 20 cc/kg normal saline, respectively). 

Seizure occurrence during first day of hospitalization can be as a result of the administration of large volume of relative hypotonic solution in patients with severe hypernatremica, and consequently rapid fall of serum sodium levels. 

In another study (12) on 97 patients with hypernatremic dehydration, the mean serum sodium level was 164.5 mmol/L at admission. Cerebral edema occurred in 49 patients during hospital staying. It was detected that over-rapid rate of fluid administration consisted of initial bolus fluid therapy and severity of hypernatremia are among significant contributing factors for cerebral edema and convulsion occurrence.

Sodium is the main cation of extracellular fluid. In hypernatremia state water shifts from intracellular space to extracellular space to equal tonicity of these 2 spaces. 

It results in some degree of intracellular dehydration. Rapid correction of dehydration can disturb this equilibrium between intra and extra cellular fluid and consequently brain edemas develop (15,16). 

In another study (6), which was performed on 57 children with hypernatremic dehydration, the mean serum sodium level at admission was 165 mmol/L and the rate of dropping of serum sodium level was 0.6 mmol/L/h. Twenty-five percentof their patients had seizures during hospital staying. They found that the mean serum sodium level at admission in these patients was higher than others, (172 mmol/L vs. 163 mmol/L, respectively; p=0.068). Also, rate of dropping of serum sodium level was higher (0.63 mmol/L/hr vs. 0.48mmol/L/h; p=0.08). They also found that there was not any relationship between rate of serum sodium dropping and complications of hypernatremia including seizure occurrence. 

In another study (14) on adult patients with dysnatremia in emergency department of a teaching hospital in Switzerland, 74 patients had severe hypernatremia, and their mean serum sodium level at admission was 152 (150-177) mm0l/L. Thirty eight percent of patients showed neurologic symptoms, and in whom serum sodium levels was significantly higher than those hypernatremic patients without any neurologic manifestations (153 vs. 151 mmol/L, respectively; p=0.02). It was revealed that rate of serum sodium level changes was not significantly different in group with neurologic manifestations and the other group without any neurologic manifestations. Also, relative slower rate of sodium correction was detected in hypernatremic patients with neurologic manifestations. 

Similarly, in our study, it was revealed that the mean serum sodium level at admission was higher in the case group compared to the control group. It was 164.3 (151-182) in case group and 162.8 (148-207) in control group (p=0.68). The rate of fall in sodium level per hour was higher in case group than controls during the first 24 hours of admission (0.59 and 0.53, respectively), but it was not statistically significant (p=0.58). Also, it was detected that most of seizures occurred before hospitalization (10 patients) and only 2 patients had seizure after admission. Severe dehydration was more significantly seen among those patients who had seizures compared to those patients without seizures (38.5% vs.14%, respectively). So, it seems that in severely dehydrated patients, hypertonicity and absolute rise of serum sodium itself, can contribute to developing seizure occurrence. Also, we found that seizure occurrence after hospitalization was rare.

Rate of falling of sodium was twice higher in first 12 hours after admission in 2 patients who had seizures after hospitalization in comparison with patients without seizures (1.12 vs. 0.54, respectively). Although it was not significant statistically (p=0.09), it can be revealed that the first several hours after admission may be critical time for seizure occurrence in hypernatremic dehydrated patients.

Seizures occurrence in the first 12 hours after admission shows that possibly, high rate of fluids intake including hypotonic fluids can lead to over rapid falling of serum sodium levels during this period. 

Our study had some limitations consisting of small size of our work. 

**In conclusion, **there was not any relationship between changes in serum sodium levels and seizure occurrence in hospital in hypernatremic dehydrated children.

Most of the seizures occurred before hospitalization and severe dehydration was seen more significantly in patients who had seizure attacks.
